# Analysis of Genetic Analysis Workshop 18 data with gene-based penalized regression

**DOI:** 10.1186/1753-6561-8-S1-S43

**Published:** 2014-06-17

**Authors:** Kristin L Ayers, Heather J Cordell

**Affiliations:** 1 Institute of Genetic Medicine, Newcastle University, Central Parkway, Newcastle Upon Tyne, NE1 3BZ, UK

## Abstract

Under the premise that multiple causal variants exist within a disease gene and that we are underpowered to detect these variants individually, a variety of methods have been developed that attempt to cluster rare variants within a gene so that the variants may gather strength from one another. These methods group variants by gene or proximity, and test one gene or marker window at a time. We propose analyzing all genes simultaneously with a penalized regression method that enables grouping of all (rare and common) variants within a gene while subgrouping rare variants, thus borrowing strength from both rare and common variants within the same gene. We apply this approach using a burden based weighting of the rare variants to the Genetic Analysis Workshop 18 data.

## Background

Genome-wide association studies have identified many common variants associated with complex diseases, yet these variants explain only a small proportion of the heritability. Previous studies demonstrate that multiple rare variants (RVs) within the same gene can contribute to monogenic disorders. Availability of imputation and sequence data has sparked interest in methods for the analysis of RVs that group or collapse variants within a region, gene, or gene pathway. Burden tests collapse RVs into a single variable (such as an indicator or count) for analysis, and require the use of a minor allele frequency (MAF) threshold to define a RV. Burden-based methods such as CAST [1], GRANVIL [2], and the variable threshold method [3], ignore the effects from the common variants (CVs), which may contain additional information. The combined multivariate and collapsing [4] method allows RVs to be simultaneously analyzed with CVs in a multivariate test. Weighting methods [5] avoid the issue of defining and separating common and rare variants by placing a predefined weight on each variant; for example, one that is inversely related to the MAF.

Burden tests have high power when all causal variants have effects in the same direction, but can lose power when there are protective effects in addition to risk effects. Thus, methods, such as C-alpha [6], were introduced, which compare the expected variances of the distribution of the allele frequencies to the actual variance. Sequence kernel association test (SKAT) [7] is a generalized version of C-alpha that allows for variant weights; its successor, SKAT-O [8], optimally combines SKAT with a burden test. All these methods operate on a single gene, ignoring information contained in other genes or outside gene boundaries. Because multiple genes can contribute to disease, we propose to analyze all genes simultaneously in a penalized regression framework. Penalized regression methods can perform model selection by shrinking the size of the coefficients, driving the coefficients of markers with little or no apparent effect down toward zero. To find the subset of genes most associated with disease, we propose penalized regression of rare and common variants (PeRC), a method that groups single-nucleotide polymorphisms (SNPs) by genes, and collapses the RVs within a gene into a single variable.

## Methods

Quantitative traits can be analyzed by minimizing the sum of square residuals (RSS). Given a phenotype vector *Y *of *m *observations, and a matrix of *p *SNP genotypes *X*, we estimate our vector of regression coefficients, *β*, by minimizing:

$$RSS\left(\beta \text{|}X,Y\right)=\overset{m}{\underset{i=1}{\sum }}{\left(y_{i}-\eta_{i}\left(\beta ,X\right)\right)}^{2}$$

where *β *is our vector of regression coefficients and $\eta_{i}$, the estimated trait value, is computed as $\eta_{i}=\overset{p}{\underset{j=1}{\sum }}x_{ij}\beta_{j}$. To perform model selection in a high-dimensional problem, we maximize the negative RSS subject to a penalty that is dependent on the magnitude of the estimated parameters. Models that include many variables with large regression coefficients incur heavier penalties, and thus optimization tends to occur with sparser models that include only the variables with the greatest effects on the RSS. We maximize our objective function, the penalized RSS:

$$O\left(X,Y,\beta ,\lambda \right)=-\frac{1}{2}RSS\left(X,Y,\beta \right)-f\left(\beta ,\lambda \right)$$

where the penalty *f *is a function of the regression coefficients and penalty parameters. Many different penalty functions have been proposed, such as the L_1 _norm (or lasso), the L_2 _norm (or ridge), and the combination of these 2 norms, the elastic net [9]. The elastic net penalty may be written as:

$$f\left(\lambda ,\beta \right)=\lambda_{1}{||\beta ||}_{1}+\lambda_{2}{||\beta ||}_{2}^{2},$$

where ${||\beta ||}_{1}=\underset{j}{\sum }|\beta_{j}|$ and ${||\beta ||}_{2}=\underset{j}{\sum }\beta_{j}^{2}$ are the L_1 _and L_2 _norm, respectively, with *j *indexing variables, and $\lambda_{1}$ and $\lambda_{2}$ are fixed parameters controlling the penalty strengths. $\lambda_{2}=0$ and $\lambda_{1}=0$ correspond to the lasso and the ridge, respectively. The L_1 _norm imposes heavy shrinkage, driving the coefficient of many variables to zero, and generally includes only 1 of a group of highly correlated variables. Ridge regression results in similar coefficients for highly correlated variables. The elastic net lies in the middle, encouraging correlated variables to enter the model together.

To encourage (a) variables within a group to enter a model together and (b) sparsity between groups, we can employ the group lasso or the sparse group lasso [10]. The sparse group lasso additionally enforces sparsity within groups, and has been previously applied in genome-wide association studies for variants with MAFs >1% in the software package Mendel [11]. This penalty function may be written as:

$$f\left(\lambda ,\beta \right)=\overset{G}{\underset{g=1}{\sum }}\left[\lambda_{1}{\left(\underset{j\in g}{\sum }\beta_{j}^{2}\right)}^{\frac{1}{2}}+\lambda_{2}\underset{j\in g}{\sum }\left|\beta_{j}\right|\right]$$

where *g *is the group index for each of the *G *groups, $\lambda_{1}$ is a parameter that controls the strength of the group penalty, and $\lambda_{2}$ is a parameter that controls the strength of the sparsity penalty. Zhou et al [11] recommend setting $\lambda_{1}=\lambda_{2}$_._

In PeRC, we use a combination of the group lasso and elastic net penalties to group both RVs and CVs within genes. We first collapse/cluster the RVs within a group into a single variable to model a common effect. We can replace $\eta_{i}$ with:

$\eta_{i}\left(\beta ,X\right)=\beta_{0}+\overset{G}{\underset{g=1}{\sum }}\left(\sum_{c\in g_{c}}x_{ic}\beta_{c}+\gamma_{g}\left\{2\frac{\sum_{r\in g_{r}}x_{ir}-d_{min}}{d_{max}-d_{min}}\right\}\right)$.

$\gamma_{g}$ is the coefficient for the collapsed RVs in group *g*, while $g_{r}$ is the set of RVs within group *g *with MAF <*τ*, and $g_{c}$ is the set of common variants with MAF ≥τ in *g*. To rescale the collapsed genotype to the range 0[2], $d_{max}$ and $d_{min}$ correspond to the maximum and minimum number of RVs in group *g *that any individual possesses. Additionally, we can encourage RVs and CVs in the same gene or window to be in the model together via a group penalty. Our generalized penalty function can be written as:

$f\left(\lambda ,\beta \right)=\overset{G}{\underset{g=1}{\sum }}\left[\lambda_{1}s_{g}{\left(\underset{c\in g_{c}}{\sum }\beta_{c}^{2}+\gamma_{g}^{2}\right)}^{\frac{1}{2}}+\lambda_{2}\left(\underset{c\in g_{c}}{\sum }\omega_{c}\left|\beta_{c}\right|+r_{g}|\gamma_{g}|\right)+\lambda_{3}\left(\underset{c\in g_{c}}{\sum }\omega_{c}\beta_{c}^{2}+r_{g}\gamma_{g}^{2}\right)\right]$.

The first term groups the RVs and CVs within our region of interest; the second and third terms correspond to the elastic net and promote sparsity of the individual CVs and the collapsed RV groups. If $\lambda =\left(\lambda_{1},\lambda_{2},\lambda_{3}\right)$, then when $\tau =1,\lambda =\left(\lambda_{1},\lambda_{2},0\right)$ corresponds to a sparse group lasso, and $\lambda =\left(0,\lambda_{2},\lambda_{3}\right)$ corresponds to the elastic net. We set the weight $s_{g}$ on each group equal to $\surd \left(l_{g}/\text{max}\left(l_{g}\right)\right),$ where $l_{g}$ is the number of CVs in group *g *plus 1 to account for the collapsed RV variable. This prevents the preferential selection of large groups solely for their ability to explain a greater proportion of phenotype variance because of increased degrees of freedom. We assign individual weights to each CV, setting $\omega_{j}$$=2\surd \left(MAF_{j}\left(1-MAF_{j}\right)\right)$, as implemented in the software Mendel. This downweights rarer variants relative to CVs. We also place a weight $r_{g}$ on the rare group coefficient of $\surd \left(f_{r}\left(1-f_{r}\right)\right)$, where $f_{r}$ is the frequency of the collapsed locus. We have currently set $\left(\lambda_{1},\lambda_{2},\lambda_{3}\right)=\kappa \left(1,1,1\right)$ where *κ *controls the amount of sparsity in the model. The objective function is maximized using Newton's method and cyclic coordinate ascent. We update our coefficients at each iteration with the CLG algorithm [12]$:\beta_{j}^{n+1}=\beta_{j}^{n}-O^{\prime }\left(\beta^{n}\right)/O^{\prime \prime }\left(\beta^{n}\right),$ where *n *is the iteration number. If the proposed new value for $\beta_{j}^{n+1}$ does not improve the objective function, we halve the proposed change in $\beta_{j}$ and reattempt.

## Results

We performed gene-based analysis for the imputed data on chromosome 3, using the UCSC genome browser to locate genes. For each gene symbol, the minimum transcription start and maximum transcription end positions were adopted as the base pair position boundaries for the gene. Overlapping genes were collapsed into a single group/gene region, which will be referred to as a gene from now on. Genes with multiple positions or less than 500 base pairs were removed. The result was a list of 1026 genes, of which 1024 were present in the imputed data. Of the 1,215,399 SNPs on chromosome 3, 590,721 were within a gene, and 315,970 of the remaining SNPs had a MAF >0.01.

We analyzed chromosome 3 with a variety of methods. PeRC and Mendel were run on a data set comprised of all SNPs within genes plus the common SNPs (MAF > 0.01) outside the boundaries of any gene. In PeRC, we used *τ *= 0.005 as the cutoff threshold for the RVs. Single marker analysis was performed in PLINK [13] on all SNPs, using an additive model. Additionally, single-gene tests were performed in the R package SKAT [7], and in the software package GRANVIL with the default RV threshold of 0.05 [2]. In SKAT, we performed 3 different analyses from the Beta(MAF,a_1_,a_2_) distribution: (a) a_1 _= 1 and a_2 _= 25, the SKAT default (SKAT), (b) a_1 _= 1 and a_2 _= 1, equivalent to C-alpha (CALPHA), and (c) a_1 _= 0.5 and a_2 _= 0.5, equivalent to the weights used in Madsen and Browning (MB) [5]. Analysis was also performed in SKAT-O with the SKAT default values. The sparse group lasso was implemented in Mendel with equal group and sparsity penalties, and variant weights based on MAF. Family information was ignored for each method, and test statistic inflation as a result of familial relationships or poor quality control was corrected via genomic control.

### Diastolic blood pressure

We analyzed both real diastolic blood pressure (DBP) and the simulated DBP for replicate 1. For both the real and simulated data, DBP was first regressed on age, age × age, sex, smoking status, and use of medication, considering each time point for each individual as a separate data entry. The mean residual over all time points for each individual became the quantitative trait variable to be used in each method. The residuals for both the real and simulated trait appeared normally distributed.

### Real data

After analysis, we looked for statistically significant (after genomic control correction for inflation and Bonferroni correction for multiple testing) SNPs or genes from those methods that provided a test statistic. For the gene-based methods, the Bonferroni corrected *p *value threshold was 4.9 × 10^−5^. For the PLINK analysis, we used a significance threshold of 10^−7^. None of the methods gave any significant results, so we examined the top 5 hits for the gene-based methods and PLINK, or the models that gave us closest to 5 independent signals for PeRC and Mendel. Little concordance existed between the non-closely related methods, except for 2 genes: *ZNF35 *was one of the top hits for SKAT/SKAT-O and GRANVIL, as was *RBMS3 *for SKAT/SKAT-O and Mendel.

### Simulated data

**Figure 1:**
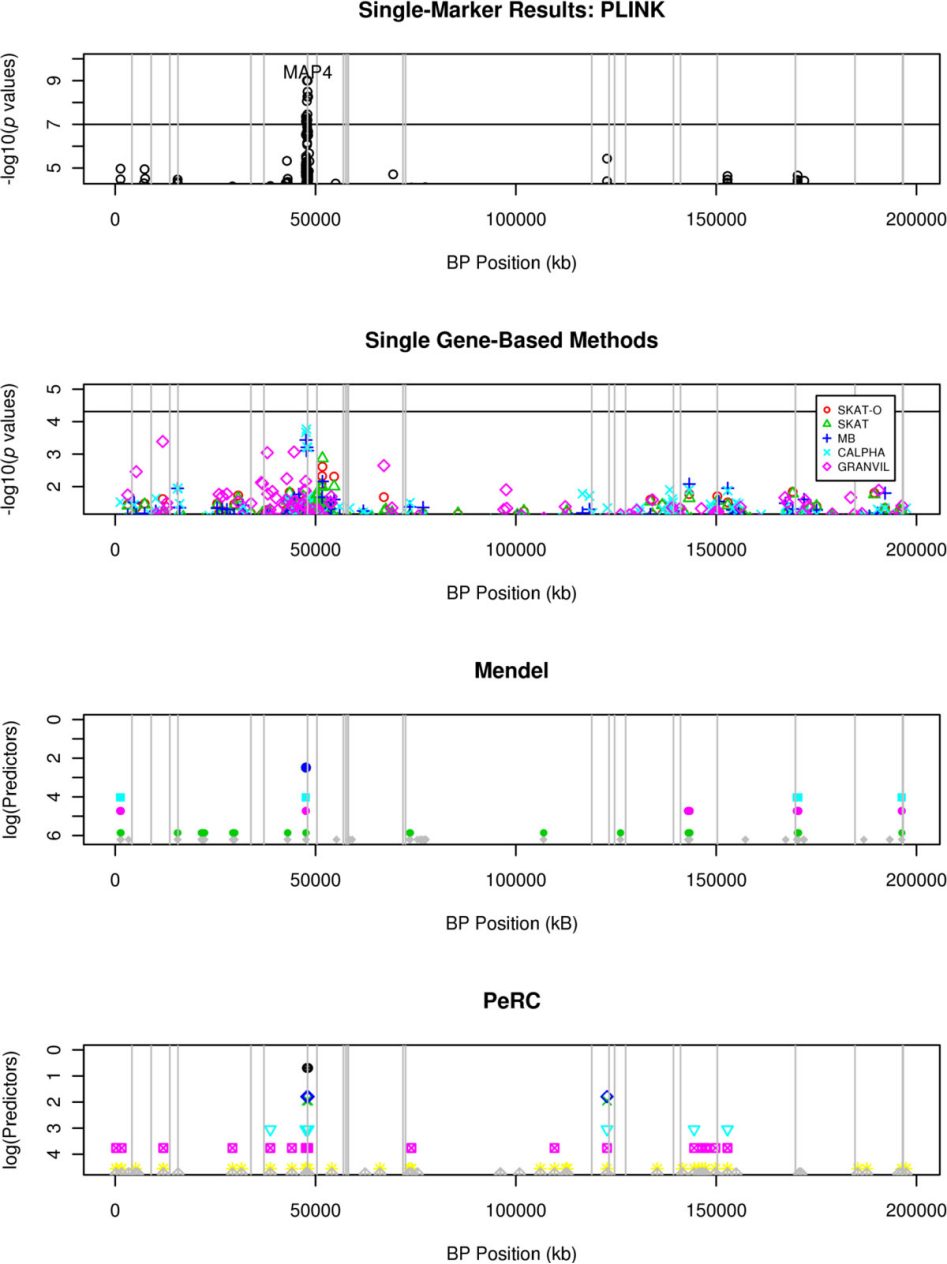
**Results for simulated DBP**. The top 2 plots report the −log_10_(*p *values) for the PLINK and the single gene-based methods, respectively. A horizontal line is drawn at the significance threshold. The points on the last 2 plots represent the predictors for Mendel and PeRC, respectively. In Mendel, the model size, or number of predictors, is selected by the user, and in PeRC, the magnitude of *κ *determines model size. Each model is represented by a different color, and the y-axis corresponds to the number of predictors in the model on the log scale. Vertical lines are drawn at the causal genes.

Figure 1 plots the results. There is a strong significant signal around *MAP4 *for the single-marker method. No genes were significant for any of the gene based methods, but *MAP4 *is a top hit for both MB and CALPHA. PeRC and Mendel both selected SNPs in the vicinity of *MAP4 *as one of their top hits. PeRC identifies the neighboring gene *DHX30 *on the left and a SNP not contained in a known gene on the right. Mendel selected the gene group *AK094639:CSPG5*, approximately 100 kilobases away.

## Conclusions

There was little power to detect effects in this data set, suggesting that most of our top findings were most likely false positives. We did observe inflation in the statistics (which we corrected using genomic control), suggesting that either (a) family structure needs to be taken into account, (b) the imputed data needs some heavy quality control, or (c) both. The Q-Q plots for the methods in the SKAT software were skewed, which may have left our corrected *p *values slightly conservative. Ideally, the imputed SNPs should have been cleaned using the imputation info score. However, several methods were still able to detect a signal near the causal gene *MAP4 *in the simulated data.

To control for family structure with imputed data, a possible strategy is to use the software package ProABEL [14] to construct the inverse variance-covariance matrix on the basis of the relationship matrix, which is made up of the kinship coefficients for family members. This package is not designed for RVs, but one could possibly collapse the counts of the RVs in a gene into a multiallelic marker to perform a burden-type test. Alternatively, we may be able to use family information in the PeRC framework by maximizing the log likelihood, $-\frac{1}{2}\left[\text{ln}\left|V\right|+{\left(Y-\beta X\right)}^{T}V^{-1}\left(Y-BX\right)\right],$ instead of the -RSS, where *V *is the variance-covariance matrix and |V| is its determinant.

## Competing interests

The authors declare that they have no competing interests.

## Authors' contributions

KLA designed the overall study, conducted statistical analyses and drafted the manuscript. HJC gave advice on methodology and analysis and assisted in revising the manuscript. All authors read and approved the final manuscript.
